# Rare Clinical Manifestation of Vasculitis

**DOI:** 10.3390/diagnostics14232623

**Published:** 2024-11-22

**Authors:** Oana-Mădălina Manole, Mihai Ștefan Cristian Haba, Iulian-Theodor Matei, Viviana Onofrei

**Affiliations:** 1 Faculty of Medicine, “Grigore T. Popa” University of Medicine and Pharmacy, 700115 Iași, Romania; manole.oana-madalina@d.umfiasi.ro (O.-M.M.); matei.theodor-iulian@d.umfiasi.ro (I.-T.M.); viviana.aursulesei@umfiasi.ro (V.O.); 2 Department of Cardiology, “St. Spiridon” County Clinical Emergency Hospital, 700111 Iași, Romania

**Keywords:** vasculitis, MINOCA, vasospastic angina

## Abstract

Background: Antineutrophil cytoplasm antibody (ANCA)-associated vasculitis usually affects small blood vessels and is characterized by the presence of circulating autoantibodies (c-ANCA or p-ANCA). The risk of cardiovascular events is threefold higher compared to general population, and cardiac manifestations include myocarditis, pericarditis, valvulitis, aortitis, or coronary arteritis. Coronary involvement is very rare, but it is a potentially life-threatening manifestation. Methods: We present an atypical cardiac scenario of p-ANCA vasculitis. Results: A 68-year-old woman with known p-ANCA vasculitis and stage 5 chronic kidney disease (CKD) on hemodialysis presented with dizziness accompanied by low blood pressure and chest pain. Electrocardiogram on arrival showed slightly ST-T changes, with negative cardiac biomarkers and no abnormalities in cardiac regional wall motion. Five hours after presentation, the patient repeated chest pain, accompanied by a drop in blood pressure and junctional escape rhythm. The highly sensitive cardiac troponin I (hs-cTnI) was raised at 560 ng/L. Coronary angiography showed coronary arteries without significant stenosis. The provocative test with intracoronary ergonovine demonstrated coronary vasospasm of the anterior descending artery accompanied by chest pain, with resolution after intracoronary nitroglycerin. Under amlodipine, nitrate, acetylsalicylic acid, statin and corticosteroids the patient did not experience the recurrence of angina. Conclusions: This case illustrates coronary involvement, manifested as coronary spasm with favorable outcomes, in systemic vasculitis. The underlying mechanism is immune-mediated inflammation in vascular walls.

## 1. Introduction

Antineutrophil cytoplasm antibody-associated vasculitis (AAV) is a subgroup of systemic vasculitis, which usually affect small blood vessels. It includes granulomatosis with polyangiitis (GPA, or Wegener’s granulomatosis), microscopic polyangiitis and eosinophilic granulomatosis with polyangiitis (EGPA, formerly known as Churg–Strauss syndrome), according to the American College of Rheumatology and the 2012 International Revised Chapel Hill Consensus Conference Nomenclature of Vasculitis [1]. First description of ANCA-associated vasculitis mechanism was proposed in 1982 by Davies and colab. [2]. AAV is characterized by the presence of circulating autoantibodies directed against the neutrophil cytoplasmic proteinase 3 (PR3 ANCA or c-ANCA) or myeloperoxidase (MPO-ANCA or p-ANCA). Microscopic polyangiitis and eosinophilic granulomatosis with polyangiitis are mainly p-ANCA positive, while granulomatosis with polyangiitis is mainly c-ANCA positive [1]. The prevalence of AAV is estimated at 46–184 per million people [3], with p-ANCA more frequent than c-ANCA [1]. AAV can affect both men and women, and the average age of diagnosis is in the sixth decade of life [1,3]. Neutrophils play a central role in the pathophysiology of AAV [4]. Overreaction of the neutrophils causes the release of lytic enzymes, metalloproteinases, neutrophil extracellular traps (NETs) and reactive oxygen species (ROS) which produce vascular endothelial injury [5]. NETs are network-like structures that contain deoxyribonucleic acid fibers, histones and antimicrobial proteins such as MPO [6]. They are produced by a process called NETosis. Recent research has demonstrated four types of NETosis: lytic, non-lytic, caspase 11/4-mediated and mitochondrial. In AAV pathophysiology, the main form of NETs production is lytic NETosis: the neutrophils perish after the release of their contents [7]. Following exposure to pro-inflammatory molecules (tumor necrosis factor alpha, interleukin 1β, C5a), neutrophils express PR3 and MPO antigens on their cell surface [8]. The binding of ANCA to PR3 and MPO triggers strong activation of neutrophils with ROS and NETs formation, increasing inflammation and endothelial injury [4]. ROS translocate MPO and neutrophil elastase from the granule nuclei with disruption of the nuclear membrane and activate phosphatidyl arginine deiminase 4 (PAD4) with arginine metabolization to citrulline and chromatin decondensation in neutrophil nuclei resulting in cell lysis. The release of NETs constitutes new sources of autoantigens for ANCA, amplifying inflammation and vascular injury in a vicious cycle [9].

The risk of cardiovascular events is 65% higher in patients with AVV, compared to the general population [10,11,12]. The highest risk seems to be during the first three months after diagnosis [13]. In addition to the traditional risk factors, such as hypertension and dyslipidemia, active inflammation and accelerated atherosclerosis increase the likelihood of cardiovascular events in patients with AAV [1,14]. Cardiac manifestations in AVV may involve any cardiac structures, resulting in myocarditis, pericarditis, valvulitis, aortitis or coronary arteritis [15,16,17]. Coronary involvement, presented as stenosis, occlusion, aneurysm or rupture of coronary arteries is extremely rare in ANCA vasculitis, but is a potentially life-threatening manifestation [18]. The pathogenesis of coronary arteritis in AVV involves immune-mediated inflammation and auto-antibody dependent reactions, with an overproduction of inflammatory cytokines, such as interferon-gamma, tumor necrosis factor-alpha and T helper (Th)-1 interleukins [17,18]. Arrythmias, conduction disturbances, angina, acute myocardial infarction, cardiac failure or even sudden cardiac death are described as clinical presentations of AAV [14,17,18,19]. Ben Brahim and colab. [19] found that cardiac involvement is more prevalent in patients with EGPA, mainly in p-ANCA positive form, as compared to c-ANCA positive GPA patients [19]. Also, it has been shown that serum MPO levels are elevated in patients with coronary arteritis, increase with severity and could be used to predict the risk for cardiovascular events [14]. Cardiac involvement in AAV is a poor prognosis factor and the cardiovascular events are the most common cause of death in these patients [17].

Myocardial infarction with nonobstructive coronary arteries (MINOCA) is defined by the presence of acute myocardial infarction based on the Fourth Universal Definition of Acute Myocardial Infarction, the absence of obstruction in any major epicardial artery based on coronary angiography, absence of clinically overt alternative cause for the acute presentation and the absence of nonischemic causes of myocardial injury [20,21]. Atherosclerosis, thrombosis and coronary artery spasm underlie the pathophysiology in MINOCA [22].

We aim to report a clinical scenario of p-ANCA vasculitis which manifested as a MINOCA with coronary vasospasm, suggesting the involvement of inflammation in coronary epicardial vessels and coronary microcirculation.

## 2. Case Presentation

A 68-year-old female treated with corticosteroids (prednisone 5 mg per day) for p-ANCA vasculitis, with stage 5 chronic kidney disease on hemodialysis (three sessions per week) and untreated secondary hypertension presented with fatigue, dizziness and low blood pressure for two previous days and episodes of constrictive chest pain occurring early in the morning.

**Figure 1:**
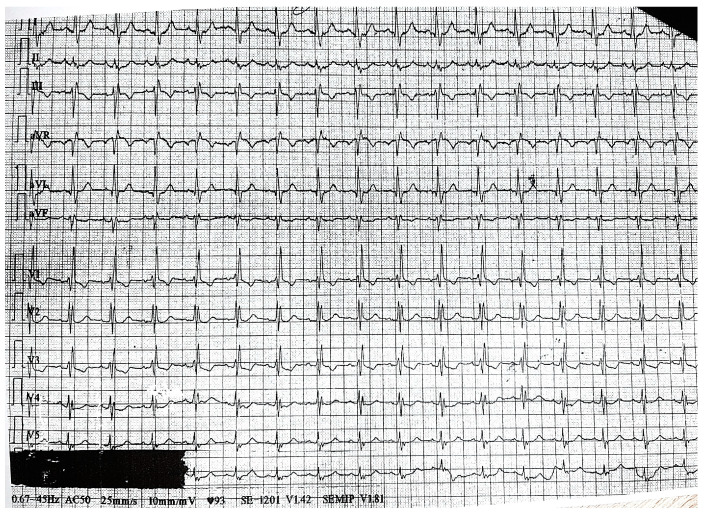
Electrocardiogram on admission showed bifascicular block and inverted T-wave in DIII, aVF, V1 and biphasic in V3.

Physical examination revealed only pale skin and mucous membranes. Vitals signs on admission were unremarkable (blood pressure during clinostatism of 108/78 mmHg, blood pressure during orthostatism of 115/80 mmHg, heart rate of 100 bpm, oxygen saturation of 98% in room air). Laboratory tests showed macrocytic anemia (hemoglobin of 7.8 g/dL, mean corpuscular volume of 99.6 fL) and inflammatory syndrome (C-reactive protein 0.7 mg/dL), with negative cardiac biomarkers (hs-cTnI less than 50 ng/L). The electrocardiogram showed right bundle branch block (previously known), left anterior fascicular block and inverted T-wave in DIII, aVF, V1 and biphasic in V3 (Figure 1). Echocardiography revealed left ventricular hypertrophy with preserved systolic function (the volumetric left ventricular ejection fraction of 55%) and no segmental kinetic disorders.

**Figure 2:**
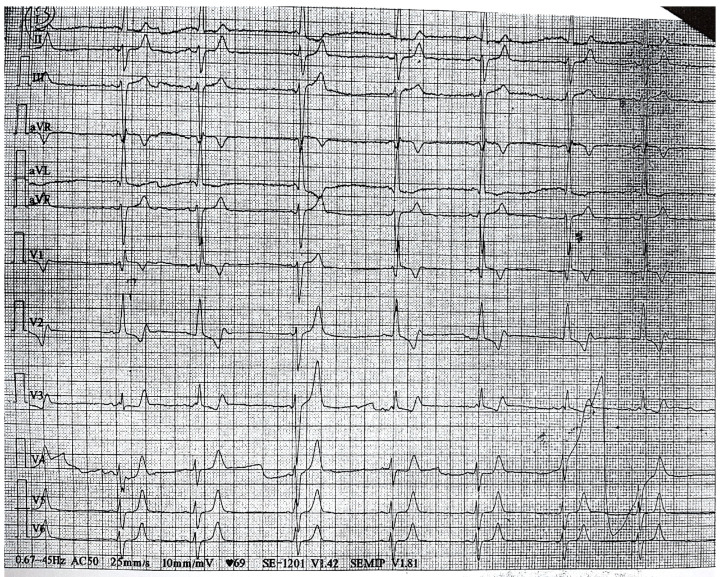
Electrocardiogram during chest pain showed junctional escape rhythm and biphasic T wave in V1–V3.

Five hours later, the patient complained of chest pain and nausea. Systolic blood pressure dropped at 70 mmHg, the pulse rate at 50 bpm and oxygen saturation was 87% in room air, requiring vasopressor, noradrenaline and oxygen therapy. The repeated electrocardiogram revealed junctional escape rhythm and biphasic T wave in V1–V3 (Figure 2), the hs-cTnI level progressively raised from 62.6 ng/L to 560 ng/L, without echocardiographic changes.

**Figure 3:**
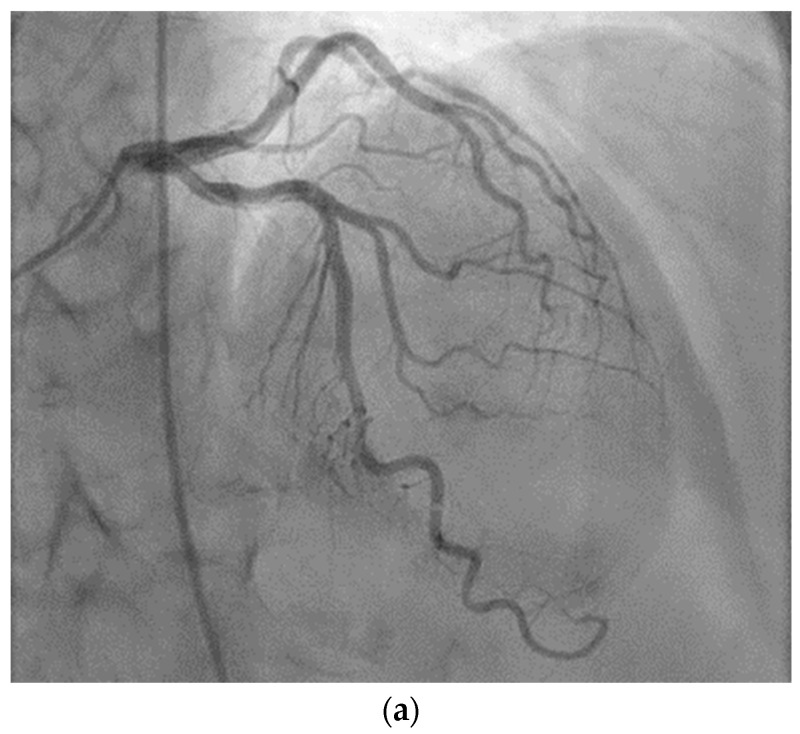

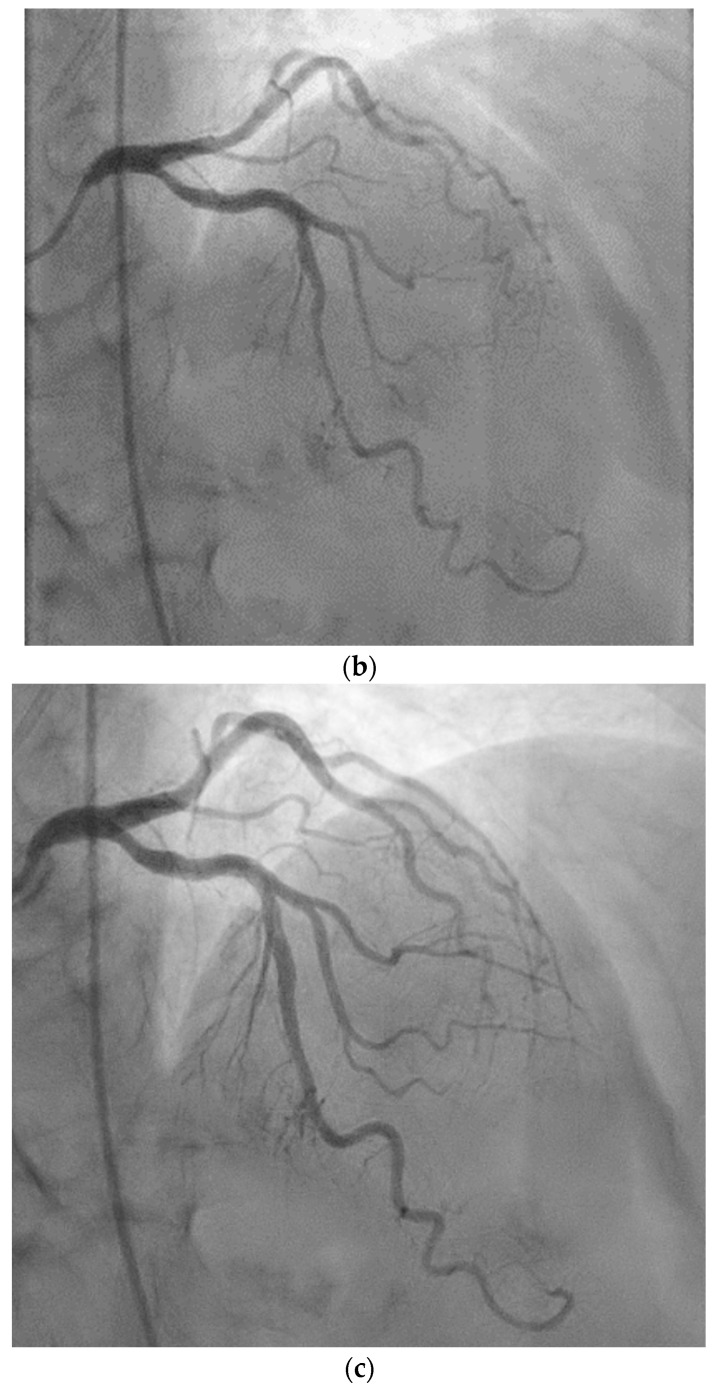
Coronary angiograms. (**a**) Before intracoronary administration of ergonovine; (**b**) After intracoronary administration of ergonovine—anterior descending artery 50–60% spasm in the proximal and medial segments; (**c**) Resolution of the vasospasm after intracoronary administration of nitroglycerin.

The coronary angiography, which showed 30–40% stenoses of the proximal and medial segments of the anterior descending artery and a 30% stenosis of the medial segment of the circumflex artery, did not justify the patient’s symptoms. It was decided to continue with intracoronary administration of ergonovine. Ergonovine was injected in incremental doses of 20, 30, 40 and 50 µg in 5–10 mL 0.9% saline solution into the left coronary artery over 2–5 min, causing a 50–60% narrowing of the proximal and medial segments of anterior descending artery accompanied by chest pain (Figure 3a–c). The rapid resolution of coronary vasospasm and chest pain occurred after intracoronary nitroglycerin administration. No further injection of ergonovine into the right coronary artery was administered.

The treatment with amlodipine, nitrate, acetylsalicylic acid and statin was started, and the patient did not have recurrence of angina during the 12-month follow-up.

## 3. Discussion

The fourth universal definition of myocardial infarction defined the acute myocardial infarction in the presence of myocardial injury (newly detected dynamic rising and/or falling pattern of cTn values above the 99th percentile upper reference limit) in the setting of evidence of acute myocardial ischemia [23]. Even if our patient had symptoms of myocardial ischemia, the first increase in hs-cTnI value (from less than 50 ng/L to 62.6 ng/L) did not meet the criteria for myocardial infarction, so we considered other causes of myocardial ischemia. Firstly, in the context of systemic blood pressure drop, the intradialytic hypotension was a proposed mechanism for myocardial ischemia. Intradialytic hypotension, a complication of hemodialysis, occurs in 20–30% of sessions [24,25], as a result of the interplay between the ultrafiltration rate, cardiac output and arteriolar tone [26]. The fast fluid exchanges between compartments overwhelm the compensatory mechanisms (myocardial contractility, heart rate, vascular tone) and decreases the cardiac output [26]. Intradialytic hypotension alone can lead to myocardial stunning in 60% of cases, which represents a reversible decrease in heart contractility caused by ischemia [25,26,27]. McIntyre and colab. [28] demonstrated the reduction in myocardial blood flow during hemodialysis. A reduction of >30% of myocardial blood flow was significantly associated with the development of regional wall motion abnormalities, which resolved after dialysis [28]. However, intradialytic hypotension occurs during hemodialysis sessions and causes myocardial stunning with reversible abnormalities in cardiac regional wall motion [25,29].

Our patient presented with a low blood pressure sixteen hours after the end of hemodialysis session and did not have any kinetic disorders in cardiac walls. Also, another explanation for myocardial ischemia could be anemia. Electrocardiogram changes in anemia include tachycardia which corelates with hemoglobin level, diffuse ST-segment depression in 50–75% of cases and T-wave changes in 29–50% of cases [30]. The underlying mechanism for ST-T complex modifications is the tachycardia, which leads to a reduced diastolic phase with subsequent decrease in blood pressure, a prominent phenomenon in acute anemia [31]. In the presented case, the anemia was chronic, secondary to end-stage chronic kidney disease and was associated with bradycardia, without diffuse ST-segment depression.

Although the increase in troponin level could be caused by chronic kidney disease, the increase in the hs-cTnI value to 560 ng/L indicated the possibility of acute myocardial infarction. It is well known that chronic kidney disease is associated with the traditional risk factors (such as hypertension and diabetes) and non-traditional risk factors (uremia-related cardiovascular disease risk factors such as inflammation, oxidative stress and abnormal calcium–phosphorus metabolism), but it is itself a major cardiovascular risk factor [32]. Patients with known chronic kidney disease are more likely to have acute myocardial infarction than stable angina, as well as a non ST-segment elevation myocardial infarction (NSTEMI) [32]. Moisi and colab. [33] showed that three-vessels or left main coronary artery disease are more often involved in patients with chronic kidney disease and acute myocardial infarction [33]. In order to exclude this, our patient underwent coronary angiography, which showed coronary arteries without obstruction, but with a coronary artery spasm on the provocative test.

Between 6% and 8% of patients with acute myocardial infarction presentation do not have obstructive coronary artery lesions on coronary angiography [20,21]. Ong and colab. [34] demonstrated that every fourth patient with acute coronary syndrome has no obstructive lesions at coronary angiography. It occurs more frequently in women, at a median age of 55 years and presents as NSTEMI [35]. MINOCA is a working diagnosis until further assessment excludes other possible ischemic or non-ischemic causes for troponin elevation [20]. This condition poses an increased risk of morbidity and mortality after diagnosis, more significantly during hospitalization and in the first 30 days after discharge [36].

One of the underlying causes responsible for MINOCA is coronary spasm [20]. In this situation, the clinical presentation varies, from chest pain that occurs at rest, often at night or early in the morning, unrelated to emotions or effort, responsive to nitrates and calcium channel blockers, to arrhythmias, cold sweating, nausea, vomiting or even syncope. Electrocardiogram changes include ST segment elevation or depression and T wave alterations [37,38]. Previous studies have proven the safety of performing coronary vasospasm provocative tests in MINOCA [38,39,40].

**Figure 4:**
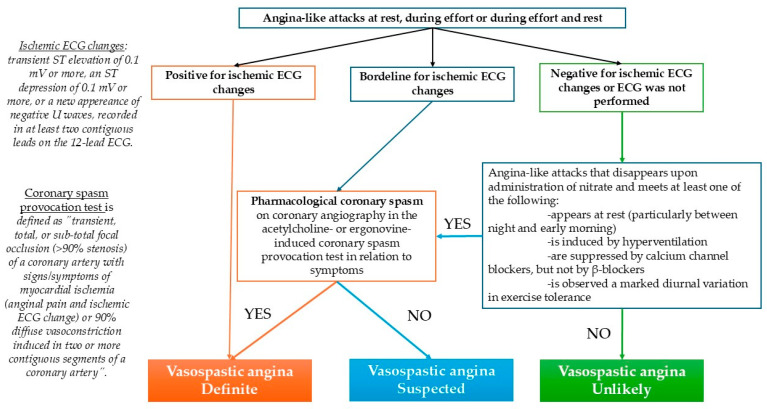
Diagnostic algorithm of vasospastic angina according to the Japanese Circulation Society (JSC)/Japanese Association of Cardiovascular Intervention and Therapeutics/Japanese College of Cardiology 2023 Guideline Focused Update on Diagnosis and Treatment of Vasospastic Angina (Coronary Spastic Angina) and Coronary Microvascular Dysfunction (adapted after [45]).

Coronary artery spasm is characterized by an exaggerated vasoconstriction of the epicardial coronary arteries and/or coronary microcirculation leading to myocardial ischemia [38]. The diagnosis of vasospastic angina can be challenging because of the transient nature of coronary vasospasm. The gold standard of diagnosing coronary vasospasm is to perform provocative testing with stimuli such as hyperventilation or with agents such as acetylcholine or ergonovine [41,42,43,44,45]. Occasionally, coronary spasm may spontaneously occur during chest pain attack while the patient is in the catheterization laboratory and ameliorates after under the intracoronary nitroglycerin [38] (Figure 4). In the presented case, it was chosen to perform the vasospasm provocation test with ergonovine because of the patient’s tendency to bradyarrhythmia, and the acetylcholine-associated risk to induce atrioventricular block when injected into the right coronary artery.

In patients with MINOCA, the coronary spasm could be documented in nearly 50% of cases [20,34]. It has been shown that was not any association between hs-cTn levels and coronary spasm [34]. More than that, it had been reported cases with normal cardiac biomarkers and spontaneous coronary vasospasm during angiography [41].

In the presented clinical scenario, in addition to ischemic symptoms and electrocardiogram changes, a less than 90% spasm of the anterior descending artery occurred along with slow flow, suggestive for microvascular spasm. It is well known that coronary microvascular dysfunction is more prevalent in women and in the presence of risk factors [38]. Other authors have reported acute coronary syndrome presentation in patients with c-ANCA [46,47,48] and p-ANCA vasculitis [16,49,50], some of them described normal major epicardial vessel with no obstructive atheroma on coronary angiography [47,49], suggesting coronary vasculitis. In the presented case, coronary vasculitis manifested as vasospasm. Several authors have described the occurrence of coronary vasospasm in patients known to have systemic vasculitis [51,52]. The proposed mechanisms for coronary vasospasm in vasculitis are endothelial dysfunction and vascular smooth muscle cells dysfunction, secondary to imbalance between vasodilators (nitric oxide and prostacyclin) and vasoconstrictors (endothelin-1 and angiotensin II) [38]. Other triggers for endothelial dysfunction have also been described, such as oxidative stress, perivascular adipose tissue properties and genetic susceptibility [38]. The involvement of Rho-kinase activation was also demonstrated [38,45]. Other authors [53,54,55] raised the possibility that coronary vasospasm had an inflammatory component. It has been found increased concentration of mast cells in the vasospastic segment of coronary arteries, so the vasospasm could be attributed to the mast cell products, histamine and leukotrienes, with well-known vasoconstrictive effect [53]. Also, a correlation between C-reactive protein levels and vasospastic angina activity has also been demonstrated [55]. More than that, several authors reported the coronary vasospasm refractory to common vasodilators, but responsive to immunosuppressive therapy, which strengthens the existence of an inflammatory component in coronary spasm [51,52,56]. These mechanisms are also implicated in coronary microvascular dysfunction, responsible for the mechanism of the clinical picture in the reported case. Ford and colab. [42] demonstrated the association between the systemic microvascular dysfunction, as that presents in vasculitis and microvascular and vasospastic angina. The underlying mechanism was also endothelial dysfunction and enhanced vasoconstriction [42], with a systemic endothelial impairment as the key mediator for coronary microvascular dysfunction [57].

The CorMicA (Coronary Microvascular Angina) trial demonstrated that personalized medical therapy guided by invasive coronary vascular function testing among patients without obstructive coronary artery disease leads to sustained angina improvement and a better-quality life at one year follow-up [42].

The current guidelines (2019 and 2024 European Society of Cardiology guidelines and 2023 JSC guideline) [23,44,45] sustained the effectiveness of calcium-channel blockers (CCB) and nitrates in coronary vasospasm. Nitrates exert vascular smooth muscle relaxing effects via nitric oxide, which increases both cGMP levels and suppresses Rho-kinase activity. CCB suppresses Ca^2+^ inflow in the vascular smooth muscle, preventing coronary vasospasm [44,45]. In 20% of cases, the coronary vasospasm is refractory to the nitrates and CCB. In this cases, other drugs were developed, such as Nicorandil, a potassium-channel opener with nitrate-like effects, Fasudil, a Rho-kinase inhibitor, or Denopamine, an adrenergic β1 receptor selective stimulator [44,45]. Acetylsalicylic acid can be used concomitantly with nitrates and CCB in coronary vasospasm, when coronary atheromatous plaques coexist. Statins have been demonstrated to reduce angina episodes by improving endothelial dysfunction and inflammatory status. Beta blockers should be avoided in the presence of epicardial spasm because they can worsen this condition [38,43,44,45].

Regarding the prognosis in coronary vasospasm, studies indicate conflicting results, but coronary implication in systemic vasculitis is an unfavorable factor [58,59].

## 4. Conclusions

This case illustrated coronary involvement, manifested as coronary spasm and microvascular dysfunction, a rare cardiac presentation, potentially life-threatening in systemic vasculitis. The probable underlying mechanism is the immune-mediation and active inflammation present in the vascular walls.

## Data Availability

The data presented in this study are available from the corresponding author upon request. The data are not publicly available due to the confidentiality of personal data.
